# Experimentally evolved Staphylococcus aureus shows increased survival in the presence of Pseudomonas aeruginosa by acquiring mutations in the amino acid transporter, GltT

**DOI:** 10.1099/mic.0.001445

**Published:** 2024-03-01

**Authors:** Ashley M. Alexander, Justin M. Luu, Vishnu Raghuram, Giulia Bottacin, Simon van Vliet, Timothy D. Read, Joanna B. Goldberg

**Affiliations:** 1 Population Biology, Ecology, and Evolution Program, Graduate Division of Biological and Biomedical Sciences, Laney Graduate School, Emory University, Atlanta, Georgia, USA; 2 Division of Infectious Diseases and Department of Human Genetics, Emory University School of Medicine, Atlanta, Georgia, USA; 3 Department of Pediatrics, Division of Pulmonary, Asthma, Cystic Fibrosis, and Sleep, Emory University School of Medicine, Atlanta, Georgia, USA; 4 Microbiology and Molecular Genetics Program, Graduate Division of Biological and Biomedical Sciences, Laney Graduate School, Emory University, Atlanta, Georgia, USA; 5 Biozentrum, University of Basel, Spitalstrasse 41,4056 Basel, Switzerland; 6 Department of Fundamental Microbiology, University of Lausanne, Quartier Unil-Sorge, 1015 Lausanne, Switzerland

**Keywords:** amino acid metabolism, cystic fibrosis, experimental evolution, interspecies competition, *Pseudomonas aeruginosa*, *Staphylococcus aureus*

## Abstract

When cultured together under standard laboratory conditions *Pseudomonas aeruginosa* has been shown to be an effective inhibitor of *Staphylococcus aureus*. However, *P. aeruginosa* and *S. aureus* are commonly observed in coinfections of individuals with cystic fibrosis (CF) and in chronic wounds. Previous work from our group revealed that *S. aureus* isolates from CF infections are able to persist in the presence of *P. aeruginosa* strain PAO1 with a range of tolerances with some isolates being eliminated entirely and others maintaining large populations. In this study, we designed a serial transfer, evolution experiment to identify mutations that allow *S. aureus* to survive in the presence of *P. aeruginosa*. Using *S. aureus* USA300 JE2 as our ancestral strain, populations of *S. aureus* were repeatedly cocultured with fresh *P. aeruginosa* PAO1. After eight coculture periods, *S. aureus* populations that survived better in the presence of PAO1 were observed. We found two independent mutations in the highly conserved *S. aureus* aspartate transporter, *gltT*, that were unique to evolved *P. aeruginosa*-tolerant isolates. Subsequent phenotypic testing demonstrated that *gltT* mutants have reduced uptake of glutamate and outcompeted wild-type *S. aureus* when glutamate was absent from chemically defined media. These findings together demonstrate that the presence of *P. aeruginosa* exerts selective pressure on *S. aureus* to alter its uptake and metabolism of key amino acids when the two are cultured together.

## Introduction

Diverse microbial communities may alter infection environments through interspecific interactions that involve metabolic exchange, altered adaptive trajectories of pathogens or symbionts, and changing antibiotic susceptibilities [1,5]. In the case of chronic infections, multiple opportunistic pathogens can coexist in their shared host environment for many generations, exerting their own respective selective pressures on one another [6]. Pairwise interactions between the opportunistic pathogens, *Staphylococcus aureus* and *Pseudomonas aeruginosa* have become a topic of interest to microbiologists, both for their importance in chronic wound infections and respiratory infections associated with the genetic disease cystic fibrosis (CF), as well as their potential to serve as a model system for studying pathogen coevolution [7,8].

Previous studies have demonstrated that the laboratory strains, *S. aureus* USA300 JE2 and *P. aeruginosa* PAO1, interact competitively when cultured together and that those interactions are greatly influenced by factors such as alginate and quinolone production by PAO1 [9,11]. Additionally, it has been observed that JE2 can adapt to secreted factors present in PAO1 supernatant in short-term evolution experiments [12]. Other studies have shown that adaptations to specific compounds such as 2-heptyl-4-hydroxyquinoline-N-oxide (HQNO) and pyocyanin may impact the antibiotic resistance profiles of either species [9,13,15]. Strain background is also a determining factor in interactions between these two species. Clinical isolates of *S. aureus* taken from CF-associated infections have been shown to have a range of tolerances to PAO1. Previous work by our group found that sensitive *S. aureus* isolates experienced as much as a six-fold decrease in recovered colony-forming units (CFUs) after coculture, while others maintained most of their population, experiencing less than a two-fold decrease in population size [16]. Additionally, other groups have found that *S. aureus* isolated from coinfections with *P. aeruginosa* have acquired mutations that reduce the efficiency of fermentative metabolism, polysaccharide production, and toxin excretion [9,17]. While many factors have been identified as influential in *S. aureus-P. aeruginosa* interactions, it is unclear how an evolving *S. aureus* population would adapt to the presence of *P. aeruginosa* in a shared environment.

In this study, we sought to gain a greater understanding for which *S. aureus* traits are under strong selective pressure when * P. aeruginosa* is introduced. To isolate and study specific genes involved in *S. aureus’* adaptation to *P. aeruginosa,* we performed our studies with the standard laboratory strains, *S. aureus* JE2 and *P. aeruginosa* PAO1. In a serial transfer evolution experiment, we showed that *S. aureus* populations could adapt to the negative selective pressures presented by *P. aeruginosa* under laboratory conditions. We found that rather than adapting to secreted toxins or contact-dependent killing, *S. aureus* improved its survival in the presence of *P. aeruginosa* by halting its uptake of aspartate through the disruption of its singular aspartate transporter, *gltT* [18]. We hypothesize that loss of function of this membrane transporter results in *S. aureus* becoming more resilient to fluctuations in nutrient availability caused by the presence of *P. aeruginosa* in its environment. These results are surprising given that *P. aeruginosa* has other well-characterized mechanisms for directly inhibiting *S. aureus* in its environment. However, our findings suggest that optimizing amino acid metabolism is a potential pathway for adaptation for * S. aureus* that co-occurs with *P. aeruginosa*.

## Methods

### Bacterial strains

Bacterial strains and plasmids used in this study are included in (Table 1). A JE2 *gltT* mutant (*SAUSA300_2329*) was obtained from the Nebraska Transposon Mutant Library (NTML) [19] and transposon was transduced into our own JE2 background. The entire *gltT* locus with the transposon was amplified from the library isolate *SAUSA300_2329* and the size of the PCR product was validated. This was performed using primers from outside of *gltT* to confirm transposon by amplicon size – 5′ AAAATTAGCCTACCTATGCAAGTTGT 3′ and 5′ TTTTGCTTTGTCATATACGTTTTCC 3′. We also used the transposon specific primer to amplify DNA from within the transposon and the gene itself using the negative strand primer – 5′ GCTTTTTCTAAATGTTTTTTAAGTAAATCAAGTAC 3′ as described by Fey *et al*. [19]. The transposon within the *gltT* gene was transduced into our parental JE2 strain using phage φ11 [20] to generate strain JE2 *gltT*::Tn, as described below.

**Table 1.** List of *Staphylococcus aureus* strains used in this study. Plasmid pGltT was made as part of this study from the pOS1-P*lgT* vector using methods described in Potter *et al*. [18]. Fluorescent plasmids pCM29 [31] and pHC48 [20] and PAO1.GFP [48] were obtained from Dr. Marvin Whiteley at the Georgia Institute of Technology

Strain	Description	Plasmid	*gltT* genotype	Reference
JE2	USA300, background for NTML	None	wild-type *gltT*	[19]
PAO1	*P. aeruginosa* strain			
PAO1.GFP	PAO1 fluorescently labelled with GFP	pMRP9-1	wild-type *gltT*	[48]
EC1	Control population 1 isolate 3 (1a_3)	None	wild-type *gltT*	This study
EC4	Control population 4 isolate 4 (4a_4)	None	wild-type *gltT*	This study
EV2	Evolved JE2, *P. aeruginosa* tolerant isolate from EE pop 1 (1 c_2)	None	1150 4 bp deletion	This study
EV3	Evolved JE2, *P. aeruginosa* tolerant isolate from EE pop 1 (1c_3)	None	951 G ->A early stop	This study
EV5	Evolved JE2, *P. aeruginosa* sensitive isolate from EE pop 4 (4c_1)	None	wild-type *gltT*	This study
EV6	Evolved JE2, *P. aeruginosa* sensitive isolate from EE pop 4 (4c_2)	None	wild-type *gltT*	This study
JE2 *gltT*::Tn	NTML transposon mutant NE566 transduced into JE2 background	None	*gltT*::Tn	[19]
JE2 *gltT*::Tn (pGltT)	JE2 *gltT* transposon mutant complemented with pGltT construct	pGltT	complemented *gltT*	This study
EV2 (pGltT)	Evolved isolate EV2 complemented with pGltT construct	pGltT	complemented *gltT*	This study
JE2.GFP	JE2 fluorescently labelled with GFP	pCM29	wild-type *gltT*	This study
JE2.DsRed	JE2 fluorescently labelled with RFP	pHC48	wild-type *gltT*	This study
JE2 *gltT*::Tn.GFP	JE2 *gltT*::Tn fluorescently labelled with GFP	pCM29	*gltT*::Tn	This study
JE2 *gltT*::Tn.DsRed	JE2 *gltT*::Tn fluorescently labelled with RFP	pHC48	*gltT*::Tn	This study

In brief, transduction was carried out by first preparing fresh φ11 lysate first with *S. aureus* strain RN4220. Collected lysate was then inoculated with NTML isolate *SAUSA300_2329* and litres were measured at 3×10^8^ plaque-forming units per millilitre. Transduction was then carried out with a multiplicity of infection of 0.1 according to methods in Krausz & Bose [21]. Transduced colonies were isolated on trypticase soy agar (TSA) (BD Difco) plates with 25 µg ml^−1^ erythromycin and confirmed by PCR, identifying the presence of the complete transposon at the correct site on the chromosome, as described above.

Fluorescently labelled strains were generated by transforming multicopy plasmids obtained from Dr Marvin Whiteley’s lab (Georgia Institute of Technology), pCM29 [22] and pHC48 [23] into both JE2 and JE2 *gltT*::Tn via electroporation [24]. This gave us the fluorescently labelled set JE2.GFP, JE2.DsRed, JE2 *gltT*::Tn.GFP and, JE2 *gltT*::Tn.DsRed (Table 1). Fluorecently labelled PAO1 [https://pubmed.ncbi.nlm.nih.gov/9535661/] was also obtained from Dr. Whiteley’s lab; here we refer to this strain as PAO1.GFP.

### Media

Cocultures for evolution experiments and subsequent confirmatory coculture experiments were conducted on TSA. After each coculture period each species was isolated on their respective isolation agar, Pseudomonas isolation agar (PIA; BD Difco) and Staphylococcus isolation agar (SIA; TSA BD Difco with 7.5 % NaCl). For liquid cultures, bacteria were cultured in lysogeny broth (LB; Teknova) which was supplemented with erythromycin (25 µg ml^−1^) to select for transposon mutants and/or with chloramphenicol (10 µg ml^−1^) to maintain fluorescent plasmids. Chemically defined media with glucose (CDMG) was made according to Hussain *et al*. [25], with varying levels of aspartate (1.1 mM or 2.2 mM) and glutamate (1.0 mM or 2.0 mM), as needed (Supplementary Methods, available in the online version of this article). CDMG was always stored at room temperature, in the dark and used within 5 days. Depleted TSB medium, used for single-cell microscopy, was prepared by diluting an overnight culture of PAO1 1 : 100 into 10 ml TSB and growing the culture for either three or 16 h. Cultures were filter sterilized (0.2 µm filter, Sarstedt) to remove cells from the supernatant and referred to in this study as ‘3hr-depleted TSB’ or ‘16hr-depleted TSB’, respectively.

### Experimental evolution

To prepare for experimental evolution, four single colonies of JE2 were picked and grown individually in 3 ml of LB media in a test tube at 37 °C in a rolling drum incubator overnight. To prepare *S. aureus* for experimental evolution we first prepared *S. aureus* populations by spotting 10 µl of an overnight culture onto 0.45 µm filters (MF-Millipore Membrane Filter) on TSA and growing them at 37 °C for 24 h. This initial growth on plates was performed to create established *S. aureus* populations that had been ‘primed’ to grow on a solid surface. Each filter was collected, and adhering cells were resuspended in 1.5 ml of LB media by vortexing for 30 s. To prepare *P. aeruginosa* for coculturing, a single colony of PAO1 was incubated in 3 ml of LB media overnight at 37 °C in a rolling incubator. The optical density 600 nm (OD_600_) of the resuspended *S. aureus* as well as the overnight PAO1 culture was measured. Cultures were normalized to the same OD by diluting the PAO1 overnight culture in LB before inoculating a coculture at a ratio of 30 : 1 (*S. aureus:P. aeruginosa*). A 30 : 1 inoculum ratio was determined through preliminary experiments to exert optimal amount selective pressure on *S. aureus* without risking a population-level extinction (approximately a four-fold decrease in population size after coculture). To account for any adaptation to culture conditions, control populations of JE2 were passaged alongside the experimental populations under identical conditions, but never cocultured with PAO1.

**Fig. 1.**
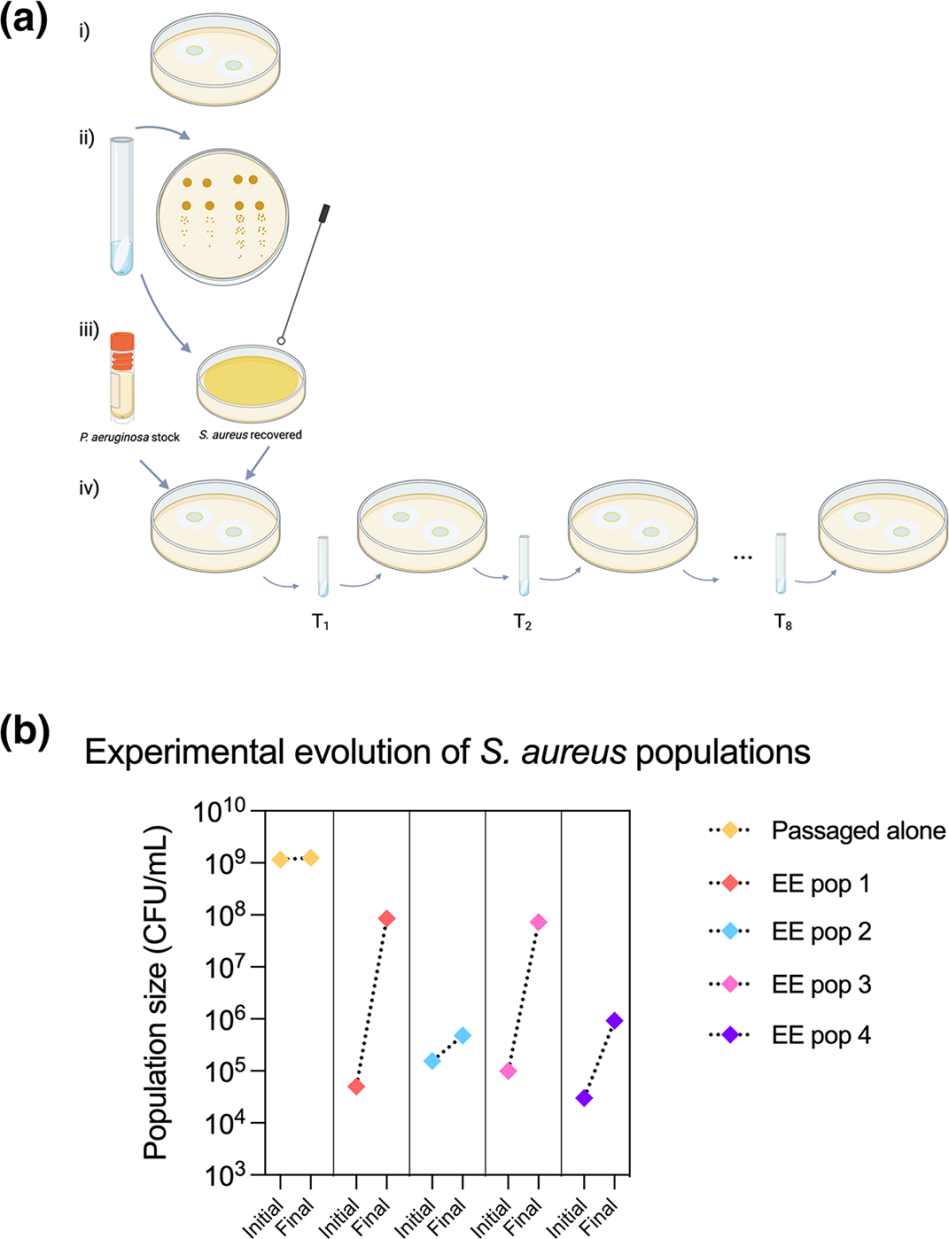
Experimental evolution with *Staphylococcus aureus* USA300 JE2 generates populations that are tolerant to *Pseudomonas aeruginosa* PAO1 in coculture. (**a**) i) Coculture setup – *S. aureus* JE2 and *P. aeruginosa*, PAO1 were cocultured for 48 h on solid TSA agar at an inoculation ratio of 30 : 1 (*S. aureus : P. aeruginosa*). A 0.45 µm filter paper was used to contain and recover the entire culture. ii) After the coculture period, filters are resuspended in liquid media and serial dilutions of the resuspension are spot plated on selective agar. After 24 h of growth, CFUs are counted for both species on their respective selective agar, SIA and PIA. iii) Then 50 µl of each resuspended culture was also plated as a lawn onto a separate SIA plate. After overnight incubation, colonies recovered from these SIA lawns were used as the source of *S. aureus* for the next coculture period, which was then incubated with a fresh overnight culture of PAO1. OD_600_ measurements were taken for both *S. aureus* and *P. aeruginosa* and the more dense was diluted to the same OD as the less dense suspension and the 30 : 1 ratio was mixed and inoculated onto filters. iv) Serial transfers are repeated for a total of eight consecutive coculture periods (**T1–T8**). Control populations are repeatedly transferred under the same conditions, but never exposed to *P. aeruginosa*. (**b**) Experimental evolution results: four populations (EE pop 1–4) were evolved in the presence of PAO1 for eight coculture periods; control population (passage alone) never exposed to *P. aeruginosa* also shown. The CFU counts from the first coculture period (initial) and eighth coculture period (final) are shown.

For the rest of the evolution experiment, coculture periods began by spotting 10 µl of (30 : 1) coculture mixtures or monocultures of *S. aureus* control populations onto 0.45 µm filters on TSA plates. All cultures were then incubated at 37 °C for 48 h. Cultures were then recovered for the next transfer by vortexing collected filters in 1.5 ml of LB media for 30 s to resuspend adhering cells. Resuspended cell mixtures were serially diluted and plated for CFUs on SIA and PIA media. Then 50 µl of resuspended coculture was also plated on SIA to be used to inoculate the next coculture. After 24 h of incubation at 37 °C, isolated *S. aureus* was then scraped off SIA plates with an inoculation loop and resuspended in LB media. This suspension was used to inoculate the next coculture period as well as to create a glycerol freezer stock of the recovered population of * S. aureus* for archiving (Fig. 1a). The evolution experiment was carried out for eight consecutive 48 h coculture periods.

### Whole genome sequencing

Single colonies were isolated from evolved *S. aureus* populations and control populations and were used to inoculate overnight cultures and subsequent glycerol freezer stocks. Isolates were struck out on SIA plates and incubated overnight at 37 °C. Cells were collected off plates the next day and resuspended in 480 µl of EDTA. *S. aureus* cells were then lysed by adding 20 µl of 10 mg ml^−1^ lysozyme and 20 µl of 5 mg ml^−1^ lysostaphin (Sigma) to the resuspended cell mixture. This mixture was then incubated at 37 °C for 1 h before proceeding with the rest of the protocol outlined for the Promega Wizard genomic DNA purification kit [26]. Genomic DNA was sequenced using the Illumina NextSeq 2000 platform at the Microbial Genome Sequencing Center (Pittsburgh, PA). Whole genome sequences were evaluated for quality using the programme FASTQC [27] and adapter sequences were removed using Trimmomatic [28]. Sequences were then screened for variants using snippy with JE2 NCBI NC_007793.1 sequence as a reference [29]. Sequences can be accessed via NCBI PRJNA1055168.

### Complementation of *gltT*

The *gltT* gene was cloned using the multicopy pOS1 shuttle vector with the constitutive P*lgT* promoter [30,31]. The *gltT* gene was amplified from wild-type JE2 using primers: 5′- AGAGCTCGAGATGGCTCTATTCAAGAG-3′ and 5′- AGATGGATCCTTAAATTGATTTTAAATATTCTTGAC-3′ and cloned downstream of the P*lgT* promoter, in the pOS1-P*lgT* plasmid, as described in Potter *et al*. [18]. The resulting plasmid, pGltT, was confirmed by whole plasmid sequencing through Plasmidsaurus (Eugene, OR) (Fig. S1). pGltT was transformed via electroporation into JE2 *gltT*::Tn, as well as the evolved isolate, EV2. Vector controls (pOS1-P*lgT*) were also generated in all three strain backgrounds: JE2, EV2, and JE2 *gltT*::Tn. All plasmids were transformed using electroporation methods of Grosser & Richardson [24].

### Population screen for variation of *gltT* in *S. aureus*

To determine the variability of *gltT,* we used a custom software, LIVID (https://github.com/VishnuRaghuram94/LIVID), which performs *in silico* PCR to extract nucleotide regions of interest from genome assemblies and compares the extracted sequence with a user-specified reference region to report mutations. LIVID was developed with similar methods as the tool AgrVATE discussed in Raghuram *et al*. [32]. For this analysis, we combined 380 assemblies from the Staphopia non-redundant diversity set [33] and 64 CF isolates [16] to create a dataset of 444 *S*. *aureus* genome assemblies. We then extracted and calculated the number of mutations in the genes *gltT, gltS, alsT, rpoD,* and *agrC,* using *S. aureus* strain JE2 (NCBI RefSeq accession GCF_002993865.1) as a reference for LIVID. To account for different *agr* groups requiring a different reference sequence, we used the software tool AgrVATE [32] to calculate the number of mutations in *agrC*. Both LIVID and AgrVATE used snippy v4.6 for identifying mutations [29]. LIVID was run with the parameters -x 1000 (minimum product size) -y 2000 (maximum product size) -d 5 (maximum allowed primer mismatch bases). AgrVATE was run with default parameters, as described in Raghuram *et al*. [32]. Mutations labelled as ‘synonymous’ were single or multi-nucleotide substitutions that did not affect the amino acid sequence. Mutations labelled as ‘AA-sequence altering’ were single/multi-nucleotide substitutions in-frame insertions/deletions that cause local changes in the amino acid sequence. Putative ‘loss of function’ variants include frameshift mutations, start-codon variants and early stops caused by non-synonymous mutations.

### Testing for *P. aeruginosa* tolerance

*S. aureus* tolerance to *P. aeruginosa* strain PAO1 was determined by coculturing *S. aureus* and PAO1 at high initial densities (>10^8^ CFUs) at a 1 : 1 ratio for 24 h and measuring recovered CFUs by serially diluting resuspended cultures and plating on SIA and PIA medias to select for *S. aureus* and *P. aeruginosa,* respectively [16]. Assays were carried out in at least five separate experiments with two biological replicates per strain.

### Murine acute pneumonia model

The impact of *gltT* activity on *S. aureus* colonization was determined in a murine acute pneumonia model. All animal procedures were conducted in accordance with the guidelines of the Emory University Institutional Animal Care and Use Committee (IACUC), under approved protocol number PROTO201700441. Eight to 10 week-old C57BL/6 female mice (Jackson Laboratories, Bar Harbour, ME) were anesthetized with a 0.2 ml mixture of ketamine (6.7 mg ml^−1^) and xylazine (1.3 mg ml^−1^) administered through intraperitoneal injection prior to infection. All mice were euthanized 24 h post-infection.

*S. aureus* strains JE2 and JE2 *gltT*::Tn were grown on SIA for 18 to 24 h at 37 °C and resuspended in phosphate buffered saline (PBS) to an OD_600_ of 8, corresponding to ~2×10^9^ CFU ml^−1^. Anesthetized mice were infected with 50 µl (~1×10^8^ CFUs) of *S. aureus* through intranasal administration (25 µl per nostril). Following euthanasia, whole lungs and nasal washes were collected aseptically. The lungs were weighed and homogenized in 1 ml of PBS (bullet blender storm 5). Homogenized lungs and nasal wash were serially diluted and plated on SIA to determine CFUs. For the murine coinfections, JE2 and JE2 *gltT*::Tn were grown on SIA for 18 to 24 h at 37 °C and suspended in PBS to an OD_600_ of 14, followed by a 1 : 2 dilution corresponding to ~4.8×10^9^ CFU ml^−1^ for each strain. Anesthetized mice were infected with 12.5 µl of culture of each *S. aureus* strain (~6×10^7^ CFU) administered sequentially and single-strain control mice were infected with either 25 µl of JE2 or JE2 *gltT*::Tn (~1×10^8^ CFU). Following euthanasia, whole lung and nasal wash were collected and processed following the procedures stated above. Serial dilutions were plated on both SIA and LA supplemented with erythromycin (25 µg ml^−1^) to determine CFUs for each strain in a coculture we subtracted the number of colonies on LA+erythromycin from the total number of colonies on SIA. Results were analysed using one-way analysis of variance (ANOVA) corrected with Šidák in GraphPad Prism nine.

### Competitive fitness assay

Strains JE2 and JE2 *gltT*::Tn fluorescently labelled with DsRed or GFP were grown individually and together in complete CDMG (1.1 mM aspartate and 1.0 mM glutamate) (+A/+G) or in CDMG with additional aspartate (2.2 mM aspartate) and no glutamate added (++A/0G). Cultures were inoculated at initial densities of OD_600_ 0.01 in flasks with 25 ml of media and incubated at 37 °C with continuous shaking for 24 h. CFUs were determined at inoculation, early growth (4–8 h after inoculation) and endpoint (22–24 h after inoculation). Both versions of each strain – JE2.GFP and JE2.DsRed and JE2 *gltT*::Tn.GFP and JE2 *gltT*::Tn.DsRed – were tested in these conditions in replicate experiments.

### Amino acid utilization

Amplite Fluorimetric l-Aspartate (Aspartic Acid) Assay Kit, and Amplite Fluorimetric Glutamic Acid Assay Kit *Red Fluorescence* (AAT Bioquest) were used to measure the concentration of aspartate and glutamate, respectively. Cell-free media was collected by filtering resuspended cocultures that were grown for 24 h on TSA plates (as was done for testing for *P. aeruginosa*-tolerance) through 0.22 µm syringe filters. Remaining nutrients were measured in the resuspended media collected from *S. aureus* monocultures and cocultures, as well as PAO1 monocultures and controls. Controls were generated by inoculating sterile filters with 10 µl of LB or 10 µl PBS and incubating for 24 h. Measurements were taken across three separate experiments, each with two replicates for each culture condition.

### Single cell imaging

Batch cultures were grown in TSB media supplemented with chloramphenicol (10 µg ml^−1^) to maintain fluorescent plasmids. Overnight cultures were diluted 1 : 100 into 3 ml fresh TSB media and grown to mid-exponential phase in a test tube at 37 °C in a shaking incubator (OD_600_ between 0.5–0.8). Subsequently, cells were washed three times with PBS to remove antibiotics and diluted to an OD_600_ of 0.1. Finally, a 1 + 1 + 1 coculture was prepared consisting of either JE2.DsRed + JE2 *gltT*::Tn.GFP + PAO1.GFP or JE2.GFP + JE2 *gltT*::Tn.DsRed + PAO1.GFP. Agarose media was prepared by adding 1.5 % agarose to either fresh or 3hr-depleted TSB or 16hr-depleted TSB.

A 1 ml droplet of agarose media was suspended between two coverslips and dried at room temperature for 30 min to create a ~3 mm thick slab, which was then cut into 5×5 mm pads. Then 1 µl of the coculture was added to the pad and dried until the liquid was absorbed. Afterwards the pad was carefully inverted and placed in a glass-bottomed dish (WillCo Wells). Six pads were added to the same dish with the following media conditions: fresh TSB, 3hr-depleted TSB, and 16hr-depleted TSB. Each condition was inoculated with one of the two strain mixtures. A small piece of water-soaked tissue was added to preserve humidity and the dish was sealed with parafilm. The experiment was repeated four times using different biological replicates on two separate days. Four replicate experiments were conducted with each labelled version JE2.GFP+JE2 *gltT*::Tn.DsRed+PAO1.GFP or JE2.DsRed+JE2 *gltT*::Tn.GFP+PAO1.GFP with each *S. aureus* strain being tested twice. Imaging of the samples began 1 h after the agar pads were inoculated.

The pads were imaged using a Nikon Ti2 inverted microscope with perfect focus system, equipped with a Hamamatsu ORCA-Flash4.0 V3 Digital CMOS camera, a Nikon NA1.42 60X Plan Apochromat phase contrast oil objective, a SPECTRA-X LED fluorescent light source, and Chroma filter sets. Images were taken every 3 min in the phase, GFP, and DsRed channels. Cells were kept at 37 °C while imaging using a climate-controlled incubator (Oko-lab).

### Image analysis

To analyse colony growth, we segmented and tracked colonies using a custom-build pipeline (code available at: https://github.com/simonvanvliet/PA-SA_Agarpads). The time-lapse movies were manually trimmed to remove later time points where cells overlapped in 3D or where excessive cell movement of *P. aeruginosa* was observed. Subsequently, images were registered using the phase_cross_correlation method of scikit-image [34]. Segmentation of colony outlines was done using the Ilastik supervised pixel classification workflow [35]. Pixels in the multi-channel image (phase, GFP, DsRed) were classified in four classes (GFP-labelled *S. aureus*, DsRed-labelled *S. aureus*, *P. aeruginosa*, and background). The classification probabilities were post-processed using custom written python code to extract individual masks for each colony. Briefly, the probabilities were smoothed with a gaussian kernel and thresholded using a fixed threshold value of 0.5. The masks were then post-processed using a binary closing operation to fill-in gaps between neighbouring cells. Finally, small objects were removed, and holes closed.

Colonies were tracked using a custom written tracking algorithm that matched colonies across subsequent frames based on the minimal center-to-center distance. Tracks were stopped when colonies merged. An automated filtering procedure was used to trim tracks whenever an unexpectedly large change in colony area was observed (indicative of missed colony merger and/or segmentation error).

Colony growth rate, *r*, was calculated as: $r=\frac{1}{∆t}\log\frac{A(T)}{A(0)}$, where *A*(0) and *A(T*) are the area (in pixels squared) of the colony at the start of the movie and after *T*=1 h, with ∆t=3 min as the imaging interval. To quantify the spatial arrangement, we calculated the minimal distance reached between the edge of the focal *S. aureus* colony to the closest pixel occupied by *P. aeruginosa*. Colonies within 300 pixels of the image frame were excluded from the analysis, as we could not accurately quantify their spatial arrangement.

## Results

### *gltT* truncation in *S. aureus* is an adaptation to *P. aeruginosa* tolerance

To monitor *S. aureus’* ability to adapt to the presence of *P. aeruginosa*, we evolved four replicate populations from four individual colonies of *S. aureus* strain JE2 under repeated exposure to *P. aeruginosa* strain PAO1. This was done by coculturing evolving *S. aureus* populations with PAO1 for 48 h on filters on TSA plates. Using filters allowed the entire coculture to be recovered after incubation. Coculture filters were then resuspended in LB and the number of *S. aureus* surviving PAO1 treatment after each coculture period was determined by plating onto selective agar and used for the next coculture experiment with fresh PAO1 (Fig. 1a). We chose to conduct this experiment with reference strains of *P. aeruginosa* and *S. aureus* (PAO1 and JE2, respectively) for which ordered transposon-mutant libraries are available so that we could easily confirm our results and test for the fitness effects of any mutation that arose in non-essential genes.

Serial transfer experimental evolution yielded populations of *S. aureus* with improved survival in the presence of *P. aeruginosa* strain PAO1. At the completion of eight repeated 48 h coculture periods, two out of four experimental *S. aureus* populations had increased their relative survival three-fold compared to the JE2 ancestral strain. Evolved populations, EE pop 1 and EE pop 3, both increased in the number of recovered CFUs by more than three orders of magnitude, with about 10^5^ CFUs being initially recovered to about 10^8^ CFUs recovered after eight serial transfers (Fig. 1b). Similar results were observed in replicate evolution experiments.

**Fig. 2.**
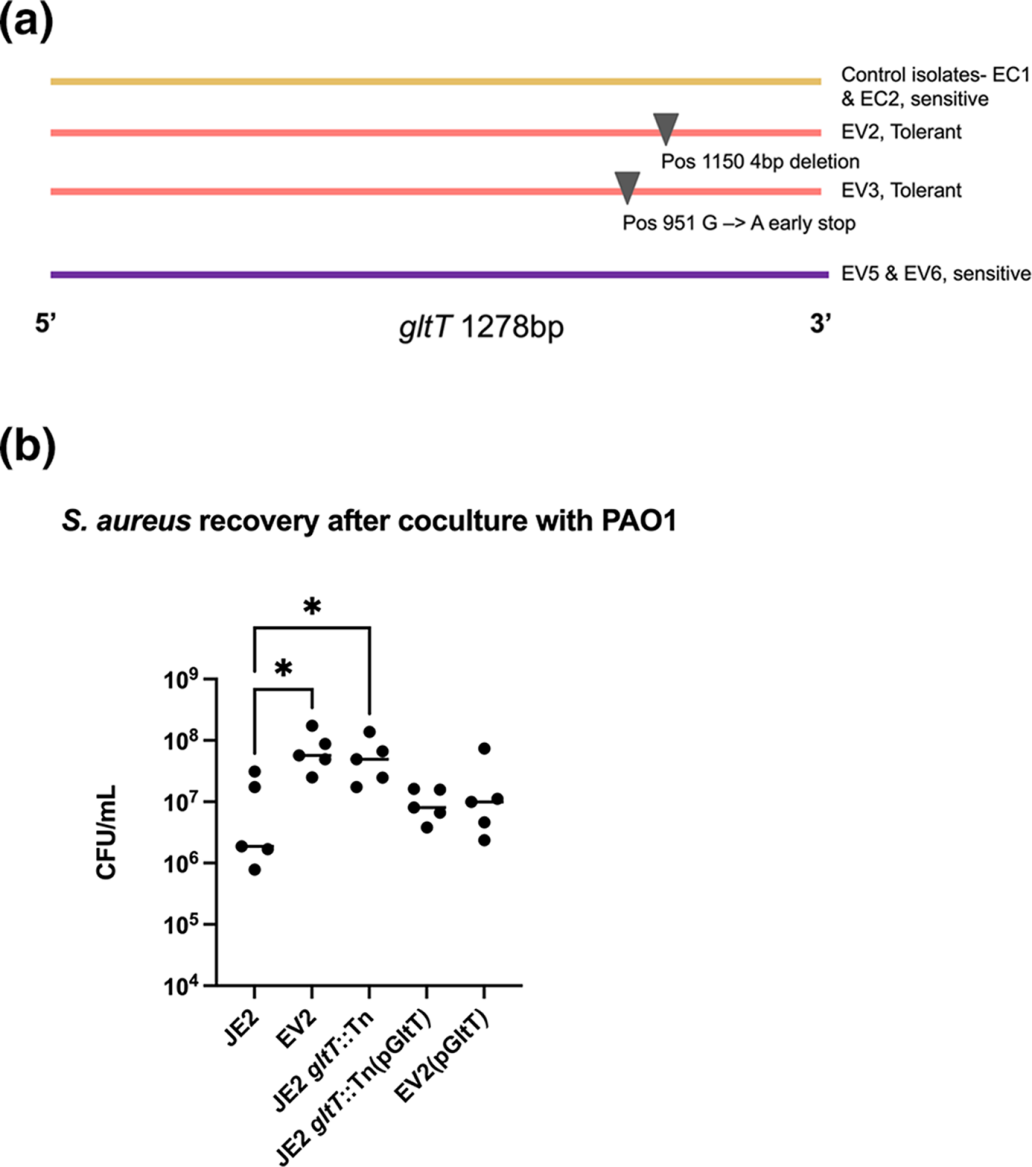
*gltT* truncation enhances *S. aureus* recovery after coculture with PAO1. (**a**) Whole genome sequencing reveals two independent truncations of the aspartate transporter, *gltT*, in sequences of two single colony isolates EV2 and EV3 taken from the same evolved *P. aeruginosa-*tolerant population – EE pop 1 (Fig. 1c). (**b**) Coculture experiments revealed that the evolved phenotype of *P. aeruginosa* tolerance was replicated in the *gltT* transposon mutant JE2 *gltT*::Tn and evolved isolate EV2. Wild-type *P. aeruginosa* sensitivity is restored in the complemented transposon mutant JE2 *gltT*::Tn(pGltT) and complemented evolved isolate EV2(pGltT). Friedman’s test for multiple comparisons yielded significant *p*-values (*) of 0.0127 and 0.0205 when comparing JE2 CFUs to EV2 and JE2 *gltT*::Tn, respectively.

Two single-colony-isolates each from an evolved *Pseudomonas-*tolerant population (EE pop 1), an evolved *Pseudomonas-*sensitive population (EE pop 4), and control population isolates that were transferred in parallel but never cultured with PAO1 were obtained. The phenotype was confirmed for these single colony isolates by screening for their tolerance to PAO1 in a 1 : 1 24 h culture on solid TSA agar (Fig. S2A). These strains were subjected to whole genome sequencing which revealed that in the evolved-tolerant isolates, EV2 and EV3, there was only one gene that contained mutations that did not appear in the sequences of the evolved but still sensitive isolate, or control population isolates. We observed that each of the evolved-tolerant isolates, EV2 and EV3, had an independent putative loss of function mutation in the gene encoding for the *S. aureus* amino acid transporter, *gltT*. In isolate EV3, a single nucleotide base substitution G → A introduced an early stop; in isolate EV2, a four-base-pair deletion resulted in a frameshift mutation (Fig. 2a). Both mutations occurred between 800–1200 bp downstream of the start codon and were predicted to truncate the protein by disrupting the 3′ portion of the coding region. All sequences can be found at NCBI PRJNA1055168. Both isolates displayed a *P. aeruginosa-*tolerant phenotype compared to their common ancestor, JE2 (Fig. S2B). GltT has been previously described as being the sole aspartate transporter in *S. aureus* that also interacts with glutamate [18,36, 37]. EV2 was selected to be used in further experiments as the comparative evolved isolate.

To confirm the linkage between *gltT* disruption and *P. aeruginosa-*tolerant phenotype, we obtained the *gltT* mariner transposon knockout mutant, *SAUSA300_2329* from the Nebraska Transposon Mutant Library (NTML) [19]. We transduced the mutation to the ancestral JE2 background, and confirmed the genotype of this strain, which we now refer to as JE2 *gltT*::Tn, had improved CFU recovery after coculture with PAO1 compared to its parent (Fig. 2b). These results confirm that the *gltT* disruption was responsible for the enhanced fitness of *S. aureus* in coculture with PAO1. When the *gltT* mutants were complemented *in trans* with the wild-type *gltT* gene on the plasmid pGltT, the PAO1 sensitivity phenotype was restored to both the JE2 *gltT*::Tn, and the evolved isolate EV2 (Fig. 2b).

### JE2 *gltT*::Tn outcompetes wild-type *S. aureus* JE2 in CDMG without glutamate

**Fig. 3.**
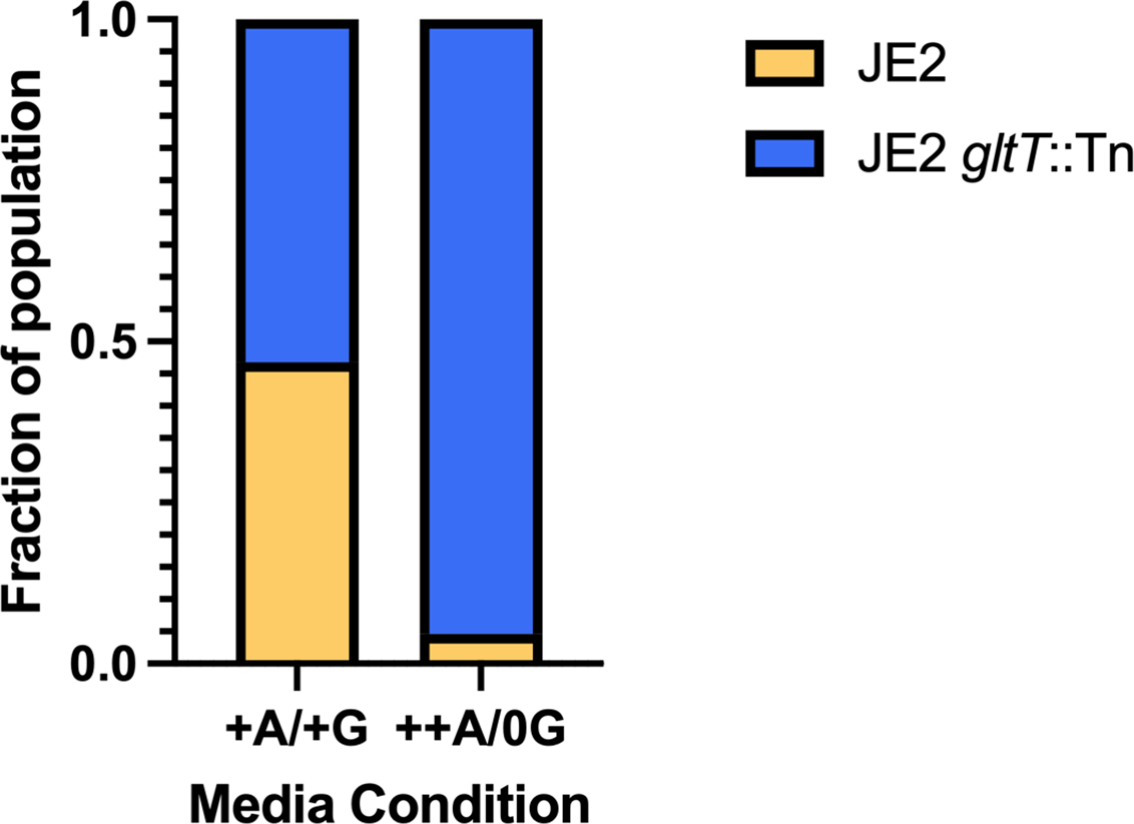
JE2 *gltT*::Tn outcompetes wild-type JE2 in CDMG when glutamate is limiting and aspartate is in excess. Fluorescently labelled strains: JE2.GFP, JE2.DsRed, JE2 *gltT*::Tn.GFP, and JE2 *gltT*::Tn.DsRed were used to test for competitive fitness in CDMG conditions. One GFP labelled strain and one DsRed labelled strain were cultured for 24 h in CDMG media alone and in coculture. Incubated cultures were then serially diluted and incubated overnight at 37 °C before CFUs were counted and number of red and green colonies recorded. Each combination strains were tested at least once across three biological replicates. In the complete CDMG condition with aspartate and glutamate in normal amounts (+A/+G) JE2 and JE2 *gltT*::Tn each made up about half of the measured CFUs in coculture. JE2 *gltT*::Tn made up the majority of CFUs recovered after 24 h in CDMG when aspartate (A) was in excess (++), and glutamate (G) was limited (0G) (++A/0G) Chi-square *p*-value<0.0001.

Growing strains individually in rich LB media (Fig. S3) yielded no significant growth differences associated with *gltT* disruption. However, we hypothesized that *gltT* mutants may be able to outcompete wild-type *S. aureus* under certain conditions. To test this, we conducted competition experiments with mutant and wild-type strains fluorescently labelled with either GFP or DsRed multicopy plasmids – pCM29 and pCH48, respectively. Labelled strains were inoculated in complete CDMG that had final concentrations of 1.1 mM aspartate and 1.0 mM glutamate (+A/+G) or in CDMG with double the amount of aspartate (2.2 mM aspartate) and no glutamate added (++A/0G). The CFU counts showed that JE2 and JE2 *gltT*::Tn were equally fit when grown in complete CDMG, with each strain making up about half of the total culture density. All cultures grew to similar final densities, >10^8^ CFUs ml^−1^. However, when grown together with excess aspartate and no glutamate (++A/0G), JE2 *gltT*::Tn outcompeted wild-type JE2; JE2 only made up about 5 % of all CFUs recovered from this growth condition (Fig. 3). When grown in isolation (not in coculture) both wild-type and *gltT* mutant strains maintained similar growth rates and yields under all conditions tested (Table S1).

### Growth rate differences, as measured by single cell microscopy, are not responsible for the *P. aeruginosa*-tolerant phenotype

**Fig. 4.**
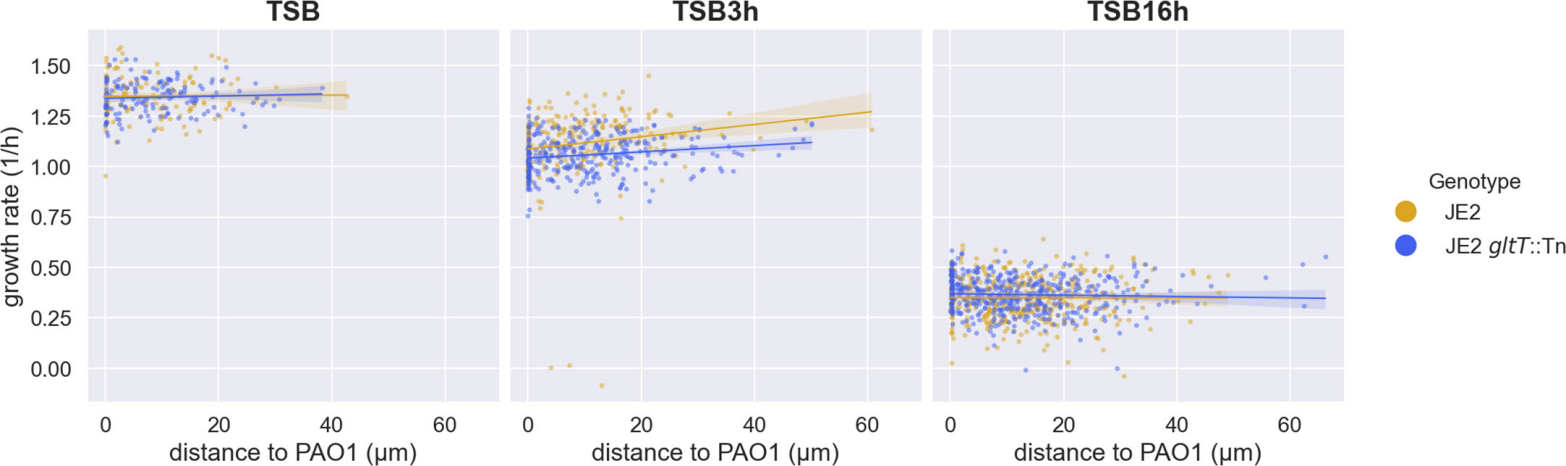
Growth rates of wild-type JE2 and JE2 *gltT*::Tn plotted against distance to nearest PAO1 cell. Fluorescently labelled strains: JE2.GFP and JE2 *gltT*::Tn.DsRed or JE2.DsRed and JE2 *gltT*::Tn.GFP were cultured with PAO1 on agar pads made from rich Trypticase Soy Broth (TSB) and 3hr-depleted TSB (TSB3h) and 16hr-depleted TSB (TSB16h). All depleted media was made by culturing PAO1 monocultures for the designated time and then collecting cell-free media. Growth rates were measured using single cell microscopy image analysis. Wild-type JE2 growth advantage over mutant JE2 *gltT*::Tn slightly increases with distance to PAO1 in 3hr-depleted TSB. However, strains grow generally at the same rate in all other conditions. Data was collected over four replicates for each of the two strain combinations. We did not observe consistent differences between the different DsRed or GFP strain combinations and their data was thus pooled. PAO1.GFP also carried a GFP label and was easily distinguished based on cell shape. The two-way ANOVA revealed significant effects of media (F(2, 18) = 11 800.29, *P*<0.001) and genotype (F(1, 18) = 4.37, *P*=0.036), as well as a significant interaction between media and genotype (F(2, 18) = 17.55, *P*<0.001) on growth. Post-hoc Tukey’s HSD test indicated significant differences between different media conditions (*P*<0.05), suggesting that the growth varied significantly depending on the media used. Additionally, significant differences were observed between genotypes in the 3hr-depleted TSB (*P*<0.05).

To test whether growth rate differences in the presence of PAO1 could be observed microscopically, we conducted single cell image analysis on cocultures with equal starting ratios of JE2, JE2 *gltT*::Tn and PAO1 and GFP and DsRed fluorescently-labelled strains. Despite the evidence we observed from the CFU data, we did not observe measurable growth differences between the two strains when grown in the presence of PAO1. We also hypothesized that growth rate differences may only be apparent in depleted media conditions. However, even in 16hr-depleted TSB (collected from a 16 h culture of PAO1), JE2 *gltT*::Tn did not have an observable difference in growth rate compared to wild-type JE2 (Fig. 4). Moreover, we did not find a dependence of *S. aureus* growth rates for either wild-type JE2 or JE2 *gltT*::Tn based on their proximity to PAO1 colonies. In fact, the only growth difference observed was a slight advantage for wild-type JE2 in 3hr-depleted TSB. Both strains had very low growth rates in 16hr-depleted TSB.

### *gltT* disruption alters amino acid uptake in *S. aureus* strains

**Fig. 5.**
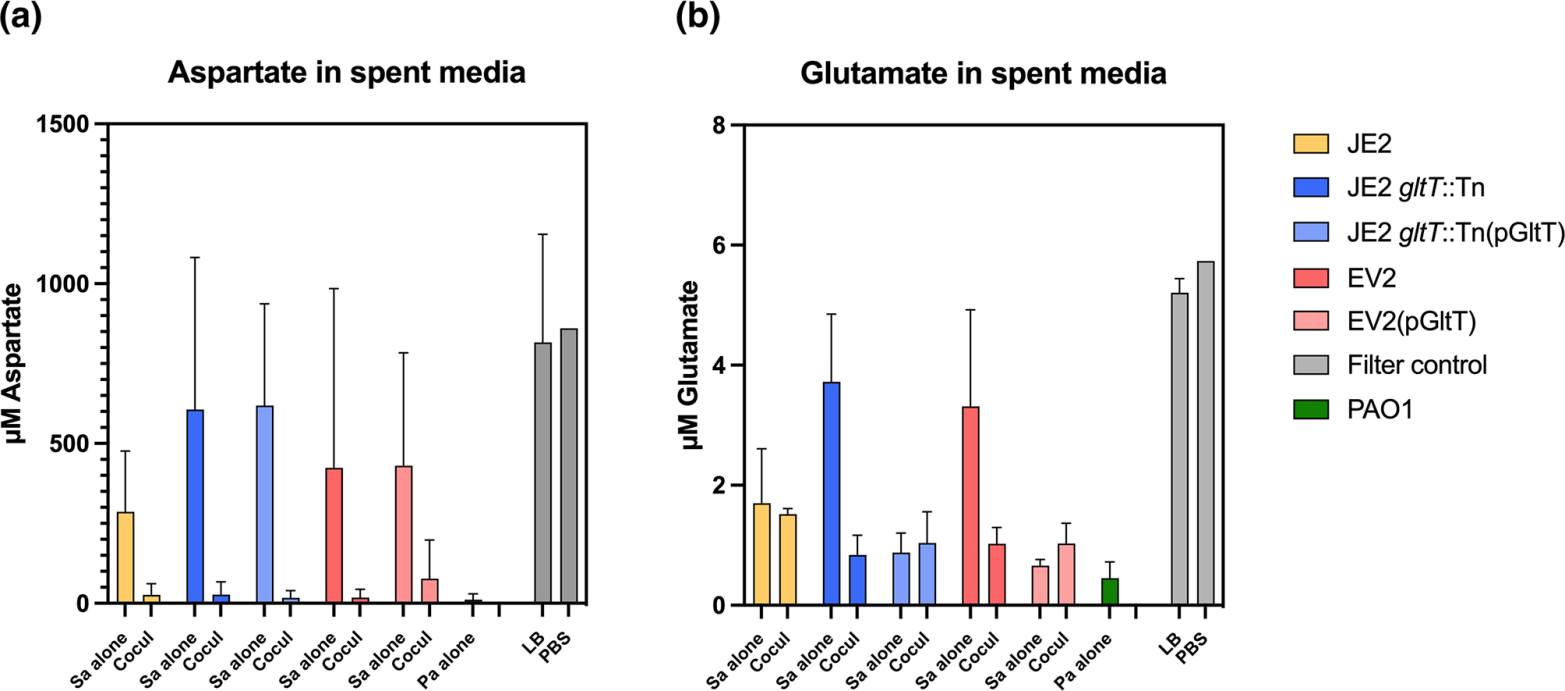
*gltT* disruption reduces *S. aureus* glutamate uptake; aspartate and glutamate are greatly reduced when PAO1 is present. Remaining aspartate and glutamate levels after 24 h of culturing on 0.45 µM filters on TSA plates, were measured using Amplite Fluorimetric kits. Cocultures with PAO1 (Cocul), *S. aureus* monocultures (Sa alone), and *P. aeruginosa* monocultures (Pa alone), were tested. Control conditions were tested by inoculating filters with 10 µl of LB media or 10 µl of PBS.

We measured concentrations of aspartate and glutamate in cell-free resuspended media that was collected from 24 h TSA cultures of the following *S. aureus* strains cultured alone or in the presence of PAO1: JE2, JE2 *gltT*::Tn, EV2 and complemented strains JE2 *gltT*::Tn(pGltT) and EV2(pGltT). These data revealed that aspartate was in higher abundance than glutamate on filters incubated on TSA. Aspartate concentration remained highest in all culture conditions where PAO1 was absent (Fig. 5). In the case of glutamate, there was very little of the amino acid remaining in any culture condition with wild-type or *gltT* complemented *S. aureus* strains (<10 µM). Both amino acids were present at higher concentrations in the resuspended media of 24 h, *S. aureus* monocultures compared that of cocultures or PAO1 monocultures (Fig. 5).

### *gltT* disruption does not impact *S. aureus* mouse respiratory colonization

**Fig. 6.**
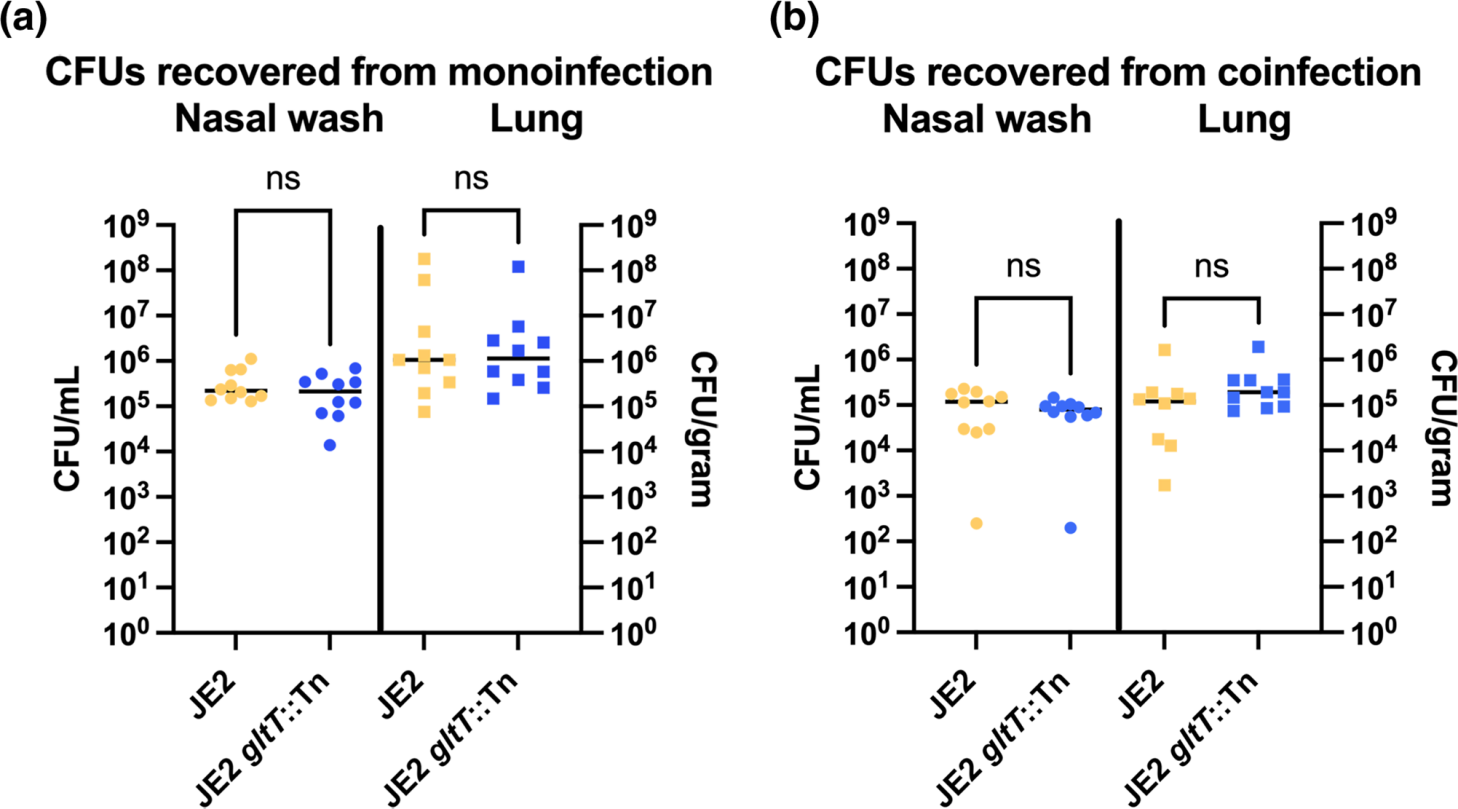
Colonization ability of *S. aureus* is not impacted by *gltT* genotype. JE2 and JE2 *gltT*::Tn are inoculated into in 8- to 10-week-old C57BL/6 female mice. For monoinfections, 1×10^8^ CFU of JE2 and JE2 *gltT*::Tn was administered intranasally. For the coinfections, 6×10^7^ CFU of both JE2 and JE2 *gltT*::Tn was sequentially administered intranasally. After 24 h post-infection, all mice were euthanized and CFUs from the nasal wash (CFU ml^−1^) and lungs (CFU gram^−1^) were recovered either on SIA (monoinfection) or both LA+erythromycin (25 µg ml^−1^) and SIA (coinfection). For coinfections CFUs of JE2 were derived by subtracting the number of colonies on LA+erythromycin from the total number of colonies on SIA. All statistics were performed using GraphPad Prism nine using one-way analysis of variance (ANOVA) with Šidák correction.

To gain an understanding of the impact of *gltT* disruption in a host environment, we carried out experiments using an acute murine pneumonia model system where mice were infected with JE2 *gltT*::Tn*,* wild-type JE2, or both strains in a coinfection. There were similar population sizes of the ancestral JE2 strain and JE2 *gltT*::Tn recovered from both the nasal wash and lung tissue for both single strain cultures and cocultures of the two strains (Fig. 6). Therefore, we concluded that *gltT* disruption did not greatly impact *S. aureus’* ability to colonize host respiratory tissues.

### *gltT* disruption was rare in diverse *S. aureus* genomes

To determine the prevalence of *gltT* mutation in *S. aureus*, we performed *in silico* PCR analysis of a set of diverse *S. aureus* genomes. Using this approach, we did not observe a significant enrichment of mutations in *gltT* when compared to other amino acid transporters in our dataset of 444 genomes. We observed 115 occurrences of non-synonymous mutations in *gltT*, with 26 distinct mutations, 25 of which were found in <10 strains. One mutation – a glutamate → aspartate change at position 891, was found in 71 strains. We compared this mutation rate to that of *gltS,* the *S. aureus* glutamate transporter, and found *gltT* to have a lower occurrence of mutations compared to this similarly functioning protein. Overall, these results suggest that *gltT* disruption is rare in *S. aureus*, even compared to other core genes encoding amino acid transporters (Table S2).

Findings from the evolution experiment and *gltT* mutant phenotyping tests indicated that *gltT* could be disrupted without severe impacts on strain fitness. We, therefore, sought to estimate the variability of *gltT* across diverse *S. aureus* lineages, including CF-associated isolates. Previous work had shown that *gltT* is a core gene and so we were able to look at *gltT* variability by screening our dataset of *S. aureus* genomes representing 380 MLST sequence types [33]. We did not find mutations identical to the ones we identified during our experimental evolution in any genomes, including CF associated isolates. Furthermore, we identified only one mutation that caused an early stop, at position 1255, truncating the protein by eight amino acids. This was the only putative non-functional mutant we observed in *gltT* and it was present in only one sample, which was not isolated from a CF-associated infection or known coinfection with *P. aeruginosa*.

## Discussion

### A newly recognized role for the *gltT* gene influencing *S. aureus* and *P. aeruginosa* interactions

**Fig. 7.**
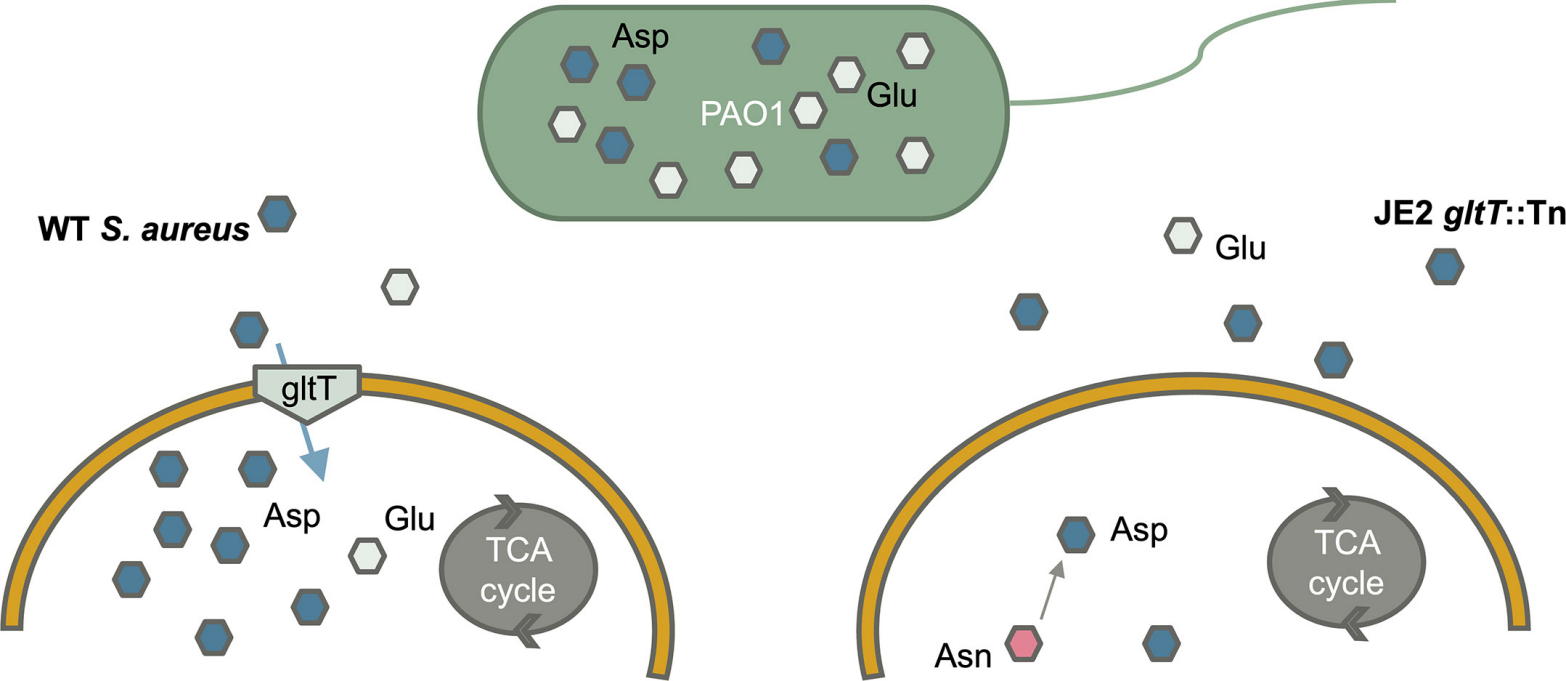
*S. aureus* adapts to the limitation of glutamate in the presence of *P. aeruginosa* by disrupting the aspartate transporter, *gltT*. Evidence gathered in our study points to this proposed model of *Pseudomonas*-tolerance by evolved *S. aureus* where wild-type JE2 (left) is effectively outcompeted by *gltT* mutant JE2 *gltT*::Tn (right) in CDMG with no glutamate and maintains large populations in the presence of PAO1. We postulate that disrupting *gltT* enables *Pseudomonas*-tolerance in strains with a disrupted *gltT* locus because they do not incur the costs of importing extracellular aspartate in a glutamate limited environment when *Pseudomonas* consumes the majority of available amino acids.

Interactions between *S. aureus* and *P. aeruginosa* have proven to be complex, and dependent on environment and strain background [12,16, 38,41]. Studies have implicated factors such as the *P. aeruginosa* mucoid phenotype, *Pseudomonas* excreted compounds or toxins, and *S. aureus* metabolic pathways such as the production of acetoin, as important factors in the interspecific interactions between *S. aureus* and *P. aeruginosa* [7,8, 16, 38, 42, 43]. Surprisingly, despite the wealth of research on interaction of these species, in our experimental system, we observed the mutation of a highly conserved gene, *gltT,* which had not previously been linked to *S. aureus-P. aeruginosa* interactions. Our findings suggest that *S. aureus* JE2 and *P. aeruginosa* PAO1 directly compete over limiting glutamate, particularly when grown at high densities on TSA plates. Our evolved isolates appear to have gained a phenotypic advantage over their JE2 ancestor by disrupting the *gltT* locus – limiting import of aspartate and glutamate and likely relying on alternative metabolic pathways. Under the conditions of the evolution experiment we designed here, there appears to be significant selective pressure for *S. aureus* to optimize its amino acid metabolism by reducing aspartate uptake potentially in response to PAO1-induced glutamate limitation (Fig. 7). Previous work has demonstrated that the *S. aureus gltT* gene is important in osteomyelitis where glutamate competitively inhibits aspartate transport through *gltT* [18]. While the osteomyelitis study above presents the inverse of the nutrient landscape that *S. aureus* is adapting to as compared to our experiment – a challenge of excess glutamate rather than it being a limiting nutrient – it also demonstrates the importance of exogenous amino acids in *S. aureus* competitive fitness and the importance of altering metabolic pathways as an adaptive strategy in changing environments. In the CF lung environment, previous work has indicated that glutamate may be freely available while aspartate is potentially limited [44]. Other studies have found that levels of glutamate and aspartate are both elevated in the CF lung and sinuses compared to non-CF lung and sinus environments [45]. It is difficult to know what micro-climates bacterial populations may find themselves in an environment with extensive surface area like the lung. It is also not known and how the presence of different species may alter the availability of nutrients for resident or invading populations. Our findings suggest that we still do not understand enough about the interaction between *S. aureus* and *P. aeruginosa* to predict the influential genes and advantageous factors in any given combination of strains and environmental conditions.

We acknowledge some limitations of our experimental evolution approach. For instance, while fresh PAO1 was introduced to each coculture period, we found that a minority population of *P. aeruginosa* were able to survive on SIA agar even though they did not form colonies. Thus we suspect that some *P. aeruginosa* cells were transferred between coculture periods and may have coevolved with the JE2 populations. However, fresh PAO1 was introduced at each coculture period, and the evolved * P. aeruginosa-*tolerant phenotypes of *S. aureus* were maintained after populations were fully isolated from any retained PAO1. Therefore, the effect of carryover *P. aeruginosa* was expected to be very small compared to the larger population of fresh introduced ancestral PAO1. Additionally, the intention with our evolution experiment design was not to exactly replicate phenomena that occur between *S. aureus* and *P. aeruginosa* in coinfections, rather we sought to observe the effect that *P. aeruginosa* has on *S. aureus* evolution in a neutral environment. The nutrient landscape of TSA is likely quite different compared to host environments as there are no selective forces from a host itself or an immune system present. Furthermore, our use of filters on agar lacks a structural environment that could allow for spatial segregation of the two species. Culturing recovery populations for 24 h on TSA agar before transferring to the next coculture period also presumably selected against slow-growing phenotypes like small colony variants, which have been observed to be associated with *S. aureus* co-occurring with *P. aeruginosa* in coinfections [46]. Note that we did not observe small colony variants in any of our evolved populations or when we monitored *S. aureus* at the single-cell level. However, in the neutral environment we facilitated on TSA plates or on agar pads in our single-cell imaging experiment, we were able to observe the effects of the strong selective pressures presented by co-occurring *P. aeruginosa* on evolving *S. aureus* populations and were able to isolate novel genetic determinants thereof.

### A model for the role of aspartate and glutamate in *P. aeruginosa* tolerance

Previous analysis of *S. aureus* metabolism has shown that glutamate derivatives are required for *S. aureus* to metabolize aspartate into oxaloacetate, a secondary metabolite required in the citric acid cycle [47]. Therefore, the absence of extracellular glutamate may lead to reduced activity of the TCA cycle (Fig. 7). This could explain why all tested *S. aureus* strains showed a reduced growth-rate when glutamate was not present in CDMG compared to CDMG with 1.0 µM of initial glutamate (Supplementary Results, Table S1). As for the role of *gltT* disruption in *S. aureus* metabolism – we did not observe significant growth rate differences between wild-type JE2 *S. aureus* and JE2 *gltT*::Tn in our CDMG monoculture assays or by single-cell microscopy. We did however, observe interesting results when looking at remaining amino acid concentrations after culturing *S. aureus* strains and PAO1 together and alone. We saw that PAO1 was more efficient than all *S. aureus* strains at consuming both glutamate and aspartate, leaving very little of either amino acid behind whenever present. Our data suggest that this may be the primary condition that selects for the disruption of *S. aureus gltT* during these coculture experimental evolution studies. The levels of remaining aspartate were higher in *S. aureus* cultures of the *gltT* mutant, which was expected due to the disruption of the main aspartate transporter. However, we also observed higher levels of glutamate remaining for *gltT* mutants, and restored wild-type phenotypes in complemented strains, suggesting that a functional *gltT* locus was essential to utilizing most of the available glutamate (Fig. 5). Interestingly, complemented strains showed similar aspartate utilization as their parent strains suggesting that *gltT* activity did not largely impact how much aspartate a strain consumed, however in general, *S. aureus* monocultures did not consume very much available aspartate. It was previously known that glutamate could be transported through *gltT* [18]*,* however, *gltS* was thought to be largely responsible for transporting the majority of glutamate [36]. Our findings here suggest that *gltT* plays a larger role in glutamate acquisition than previously thought.

These data suggest that despite its important role in amino acid metabolism, there were few apparent fitness trade-offs associated with disrupting *gltT* when strains are grown in isolation [37]. However, the finding that *gltT* mutations are extremely rare in non-laboratory-adapted strains reinforces the key metabolic role of this core gene. We postulate that continuing to import aspartate in the wild-type strain in the absence of glutamate may lead to a buildup of aspartate intracellularly and a corresponding reduced competitive fitness. This hypothesis is supported by our finding that along with its increased *P. aeruginosa*-tolerance, JE2 *gltT*::Tn was able to outcompete wild-type JE2 in the CDMG condition where additional aspartate was added and no glutamate was added (++A/0G) (Fig. 3).

Further experimentation is needed to better understand how amino acid metabolism facilitates *S. aureus-P. aeruginosa* interactions; however, we conclude here that in this evolution experiment, *S. aureus* is primarily adapting to the limitation of glutamate in its environment by disrupting its aspartate transporter and relying on alternative metabolic pathways to carry out the TCA cycle. In short, the major source of negative selective pressure that *S. aureus* experienced when grown in the presence of *P. aeruginosa* was competition over exogenous amino acids.

### *In vitro* experimental evolution is a useful tool for studying pathogens

Experimental or directed evolution experiments carried out in laboratory conditions can be a powerful way to reduce complex adaptive phenotypes in important pathogens like *S. aureus* and *P. aeruginosa* to single genetic determinants. Our findings here lay important groundwork for understanding the development of coinfections by highlighting the importance of nutrient availability in the facilitation of *S. aureus-P. aeruginosa* coexistence. Because *gltT* is so highly conserved in natural populations, the link between *gltT* and *P. aeruginosa* tolerance likely could not have been identified by studying clinical isolates alone. Our *in vitro* experiments suggest that while rare, *gltT* mutants can colonize lung tissue just as well as wild-type strains and would be more likely to coexist with *Pseudomonas* in a coinfection due to their increased tolerance of co-occurring * P. aeruginosa*. Therefore, this genotype could be important to screen for when treating coinfections and is certainly important to consider in the development of new therapies that may alter the availability of nutrients in the infection environment.

## supplementary material

10.1099/mic.0.001445 Uncited Supplementary Material 1.
