# Supplementary material for: Experimentally evolved Staphylococcus aureus shows increased survival in the presence of Pseudomonas aeruginosa by acquiring mutations in the amino acid transporter, GltT
Source: Microbiology (Reading). 2024 Mar 1;170(3):001445. doi: 10.1099/mic.0.001445 (PMC10999751; doi:10.1099/mic.0.001445)
Supplement: Uncited Supplementary Material 1. [file mic-170-01445-s001.pdf]

Supplementary Methods

Growth in chemically defined media

To test for the nutrient requirements of mutant and wild-type *S. aureus,* strains were grown in CDMG according to Hussain et al. (1991) with varying levels of aspartate and glutamate across six different conditions. The tested conditions were: 1) No asparate or glutamate added (0A/0G), 2) 1.1 mM asparate with no glutamate added (+A/0G), 3) 2.2 mM asparate with no glutamate (++A/0G), 4) 1.1 mM asparate with 1.0 mM glutamate (+A/+G), 5) 2.2 mM asparate with 1.0 mM glutamate (++A/+G), 6) no asparate with 1.0 mM glutamate (0A/+G). This array of nutrient conditions was used to test the growth of the evolved isolate (EV2), JE2 *gltT*::Tn, and ancestral JE2 growth rate (r - $hour^{-1}$) and area under the curve integral (AUC – OD_{600}xhour) were calculated using the program growthcurver (https://github.com/cran/growthcurver)(Sprouffske & Wagner, 2016). Growth assays were conducted in 96 well microtiter plates for 24 hours at 37°C with continual shaking. Growth was measured via OD_{600} measurements taken every 20 minutes (**Supplementary Table 1**).

Supplementary Results

gltT disruption does not alter *S. aureus* growth in chemically defined media

When grown alone in nutrient rich LB media *gltT* mutants, JE2 *gltT*::Tn and EV2 were not observed to have significant fitness differences compared to the parental JE2 (**Supplementary Figure 3**). We did not find statistically significant growth rate differences for any CDMG condition tested between wild-type JE2, JE2 *gltT*::Tn or *gltT* complemented strain JE2 *gltT*::Tn(pGltT). We noted that all strains grew slower in the absence of glutamate (**Supplementary Table 1**). Additionally, complemented strain JE2 *gltT*::Tn(pGltT) had a significantly lower AUC value compared to JE2 *gltT*::Tn in the conditions with aspartate and no glutamate, (+A/0G) and (++A/0G).

Strain	Chemically defined media condition					
	+A/+G	+A/0G	++A/0G	++A/+G	0A/+G	0A/0G
**JE2** (r)	1.12 ± 0.08	0.52 ± 0.19	0.53 ± 0.14	1.03 ± 0.09	0.90 ± 0.07	0.46 ± 0.15
AUC	15.72 ± 1.23	12.35 ± 2.08	11.30 ± 2.52	16.33 ± 1.97	15.48 ± 0.50	11.01 ± 4.90
**JE2 *gltT*::Tn** (r)	1.03 ± 0.11	0.66 ± 0.05	0.65 ± 0.12	1.11 ± 0.20	0.99 ± 0.11	0.59 ± 0.21
AUC	14.98 ± 1.89	15.11 ± 1.21	14.53 ± 2.48	14.95 ± 2.59	15.42 ± 2.01	13.23 ± 4.33
**JE2 *gltT*::Tn (pGltT)** (r)	1.24 ± 0.11	0.42 ± 0.10	0.59 ± 0.09	1.09 ± 0.30	0.84 ± 0.34	0.59 ± 0.13
AUC	15.95 ± 0.87	9.79 ± 3.27	9.64 ± 2.70	17.46 ± 2.15	16.69 ± 0.65	12.1 ± 3.10

Supplementary Table 1. Wild-type JE2, JE2 *gltT*::Tn, and complemented strain JE2 *gltT*::Tn (pGltT) were tested in six different chemically defined media conditions with varying levels of glutamate and aspartate to determine the effects that aspartate and glutamate had together and individually on mutant *gltT* fitness. None of the conditions tested yielded statistically significant growth rate (r) differences between any of the three strains when tested with nonparametric Kruskal-Wallis test with Dunn's correction*.* JE2 *gltT*::Tn (pGltT) had significantly lower values for AUC when compared to JE2 *gltT*::Tn in the +A/0G and ++A/0G conditions (p-values p=0.0144, and p= 0.0267, respectively - nonparametric Kruskal-Wallis test with Dunn's correction). Statistical analysis performed using GraphPad Prism 9.

Gene	Gene length (bp)	Number of mutations					
		Absolute number of mutations			1kb normalized number of mutations		
		Putative loss of function	AA sequence altering	Synonymous	Putative loss of function	AA sequence altering	Synonymous
gltT	1278	1	114	2341	0.78	89.2	1831.77
gltS	1209	4	344	5539	3.31	284.53	4416.05
alsT	1461	0	450	4457	0	308.01	3050.65
rpoD	1107	0	12	1019	0	10.84	920.51
agrC	1245	14	1504	5196	11.24	1208.03	4173.49

Supplementary Table 2. Summary of genes screened for variability across 444 diverse *S. aureus* genomes. The custom tool LIVID was used for genes, *gltT, gltS, alsT,* and *rpoD.* AGRVATE was used to screen variability of *agrC* with default parameters (Raghuram et al., 2022).

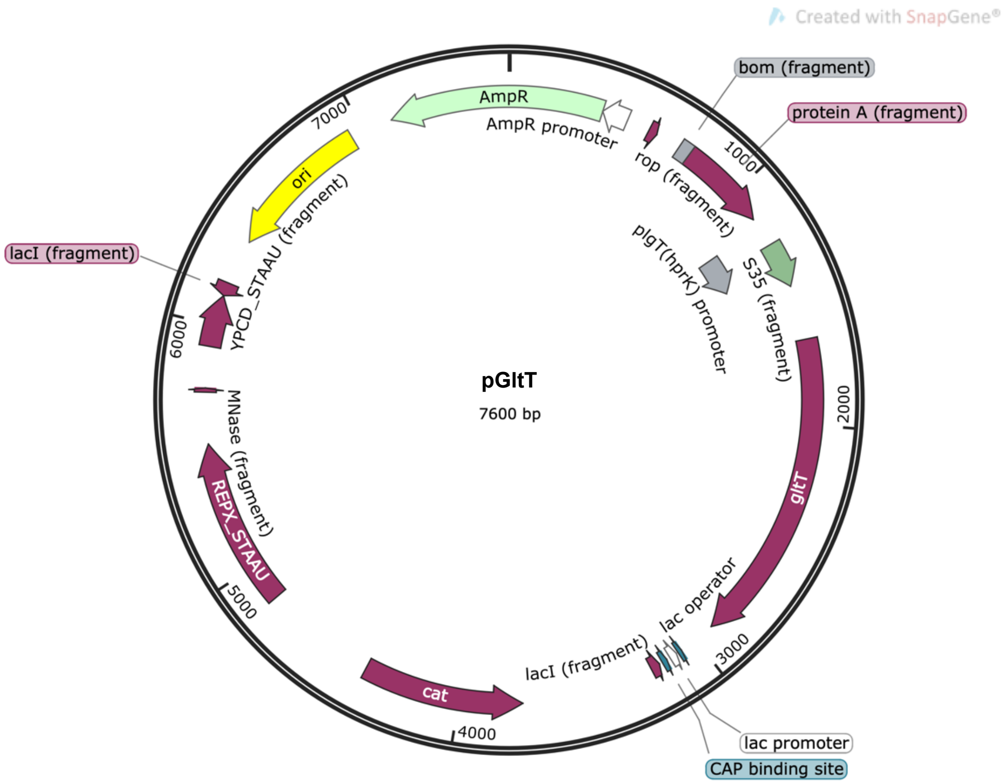

Supplementary Figure 1. Map of the *gltT* complementation vector, pGltT, using the program Snapgene®. Construct was created by cloning the JE2 wild-type *gltT* coding sequence into the multiple cloning site of the pOS1.plgT *E. coli* – *S. aureus* shuttle vector. P*lgT* promoter was found by matching plasmid sequence to the region 275 bp upstream of the *hprK* operon which includes the *lgT* gene as described in Bubeck Wardenburg et al. (2006). Primers used for amplifying *gltT* from wild-type JE2 were 5'-AGAGCTCGAGATGGCTCTATTCAAGAG-3' and 5'-AGATGGATCCTTAAATTGATTTTAAATATTCTTGAC-3' as described in Potter et al. (2020).

A B

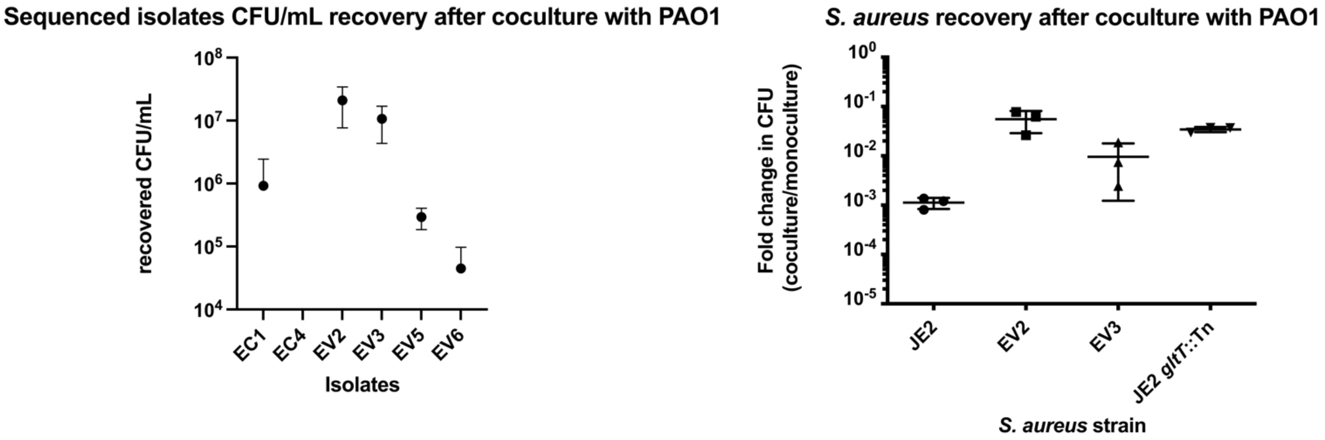

Supplementary Figure 2. A) Recovered CFUs/mL of all sequenced isolates after a 24-hour coculture period with PAO1 at a 1:1 initial ratio and initial OD_{600} of 0.01. Isolates represent single colony isolates taken from experimental or control populations that were selected for whole genome sequencing. EC1 and EC4 are control isolates from populations passaged in parallel but never cocultured with PAO1 during the evolution experiment. There were no *S. aureus* colonies recovered from EC4 when cocultured with PAO1 so therefore, there is no value plotted for this isolate. EV2 and EV3 isolates were isolated from experimental population EE pop 1 where high CFU/mL recovery is indicative of their evolved *P. aeruginosa* tolerant phenotype (Figure 1). EV5 and EV6 were isolated from experimental population EE pop 4 (Figure 1) and their lower CFU/mL recovery is representative of the population's sensitivity to PAO1's presence even after 8 serial transfers. B) Fold change in CFU/mL recovered from strains JE2, EV2, EV3, and JE2 *gltT*::Tn. Fold change is used here to control for any growth differences among strains by dividing the CFUs/mL recovered after coculture by those recovered from corresponding *S. aureus* monocultures. JE2 *gltT*::Tn shares the evolved *P. aeruginosa* tolerant phenotype with evolved isolates EV2 and EV3.

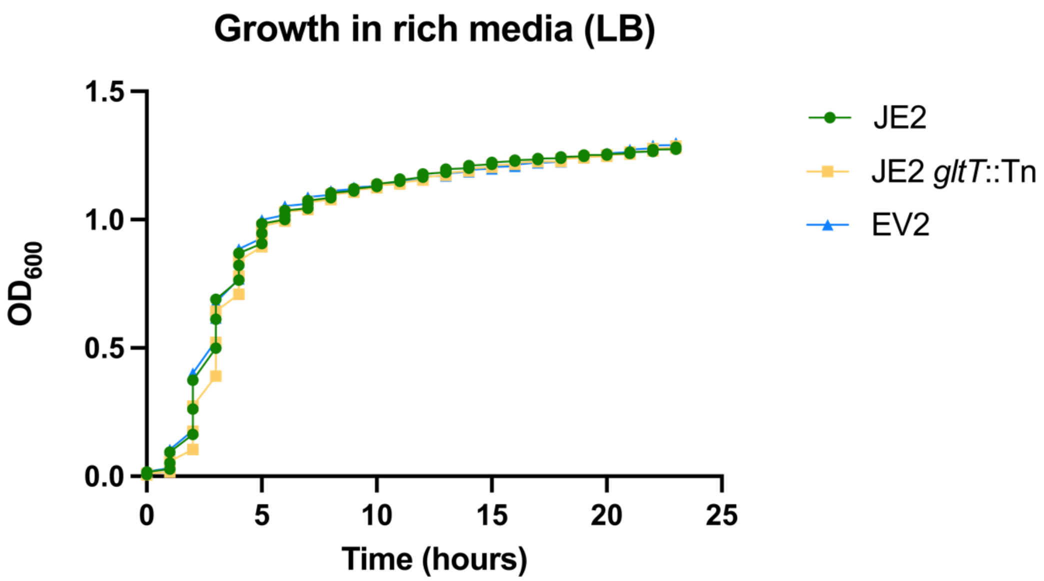

Supplementary Figure 3. Growth of *gltT* variants in rich LB media. Strains JE2, JE2 *gltT*::Tn and evolved isolate EV2 grow very similarly under nutrient rich conditions.

SUPPLEMENTARY REFERENCES

Bubeck Wardenburg, J., Williams, W. A., & Missiakas, D. (2006). Host defenses against *Staphylococcus aureus* infection require recognition of bacterial lipoproteins. *Proceedings of the National Academy of Sciences*, *103*(37), 13831–13836. https://doi.org/10.1073/pnas.0603072103

Hussain, M., Hastings, J. G. M., & White, P. J. (1991). A chemically defined medium for slime production by coagulase-negative staphylococci. *Journal of Medical Microbiology*, *34*(3), 143–147. https://doi.org/https://doi.org/10.1099/00222615-34-3-143

Potter, A. D., Butrico, C. E., Ford, C. A., Curry, J. M., Trenary, I. A., Tummarakota, S. S., Hendrix, A. S., Young, J. D., & Cassat, J. E. (2020). Host nutrient milieu drives an essential role for aspartate biosynthesis during invasive *Staphylococcus aureus* infection. *Proceedings of the National Academy of Sciences*, *117*(22), 12394–12401. https://doi.org/10.1073/pnas.1922211117

Raghuram, V., M, A. A., Qi, L. H., A, P. R., B, G. J.*, & D, R. T. (2022). Species-Wide Phylogenomics of the Staphylococcus* aureus Agr Operon Revealed Convergent Evolution of Frameshift Mutations. *Microbiology Spectrum*, *10*(1), e01334-21. https://doi.org/10.1128/spectrum.01334-21

Sprouffske, K., & Wagner, A. (2016). Growthcurver: an R package for obtaining interpretable metrics from microbial growth curves. *BMC Bioinformatics*, *17*(1), 172. https://doi.org/10.1186/s12859-016-1016-7
